# Effects of Cardiovascular Interval Training in Healthy Elderly Subjects: A Systematic Review

**DOI:** 10.3389/fphys.2020.00739

**Published:** 2020-07-31

**Authors:** Leandro de Oliveira Sant'Ana, Sérgio Machado, Aline Aparecida de Souza Ribeiro, Natália Rodrigues dos Reis, Yuri de Almeida Costa Campos, João Guilherme Vieira da Silva, Fabiana Rodrigues Scartoni, Amanda Fernandes Brown, Estêvão Rios Monteiro, Jefferson da Silva Novaes, Jeferson Macedo Vianna, Henning Budde

**Affiliations:** ^1^Post Graduate Program in Physical Education, Federal University of Juiz de Fora, Juiz de Fora, Brazil; ^2^Sport and Exercise Science Laboratory, Catholic University of Petrópolis, Rio de Janeiro, Brazil; ^3^Laboratory of Physical Activity Neuroscience, Salgado de Oliveira University, Rio de Janeiro, Brazil; ^4^School of Physical Education and Sports, Federal University of Rio de Janeiro, Rio de Janeiro, Brazil; ^5^Faculty of Human Sciences, Medical School Hamburg, University of Applied Science and Medical University, Hamburg, Germany

**Keywords:** aerobic training, autonomic response, hemodynamic response, blood pressure, healthy elderly

## Abstract

The aim of this review is to demonstrate the effects of cardiovascular interval training (IT) on healthy elderly subjects. We used the recommendations of the Preferred Reporting Items for Systematic reviews and Meta-Analyses (PRISMA) guidelines. The following variables were observed: resting heart rate (HR), systolic blood pressure (SBP), diastolic blood pressure (DBP), mean arterial pressure (MBP), heart rate variability (HRV), baroreflex activity (BA), and maximal oxygen uptake (VO_2max_). Studies were searched for in the MedLine, PubMed, and Sport Discus databases considering publications between 1990 and 2019. To find the studies, the keywords used were “Interval and Elderly Training” or “Interval Training and Baroreflex Sensing” or “Interval Training and Aging and Pressure Arterial and Blood Pressure Training” or “Interval Training and Variation in Aging and Heart Rate” or “Interval Training and Sensitivity to the Elderly and Baroreflex” or “Interval Training and Variability in the Elderly and Heart Rate.” The systematic search identified 1,140 hits. The analysis of the study was performed through a critical review of the content. One thousand one hundred forty articles were identified. Of these, 1,108 articles were excluded by checking the articles and abstracts. Finally, 32 studies were selected for full reading while 26 studies were eliminated because they did not contain a methodology according to the purpose of this review. Thus, six studies were included for the final analysis. The PEDro score was used for analyzing the study quality and found 4,8 ± 1,3 points (range: 3–6). Positive results were found with the different IT protocols in the observed variables. Results show that IT protocols can be an efficient method for functional improvement of cardiovascular and cardiorespiratory variables in the healthy elderly, especially HR, SBP, DBP, MAP, HRV, BA, and VO_2max_. However, this method can be included in the prescription of aerobic training for the elderly to obtain conditional improvements in the cardiovascular system, thus being an important clinical intervention for the public.

## Introduction

According to the World Health Organization, the number of elderly people has been increasing over the years (World Health Organization, 2016). The American College of Sports Medicine (ACSM) and the American Heart Association (AHA) jointly directed positions on the importance of physical training in improving cardiovascular and cardiorespiratory fitness in the face of aging (Nelson et al., 2007). Physiological changes in aging are inevitable (Adamson et al., 2019) but can be partially prevented by the practice of cardiovascular (Soares-Miranda et al., 2014) and cardiorespiratory exercise (Lepretre et al., 2009). Aging promotes severe changes in the cardiovascular system (Molmen et al., 2012). Disorders related to the cardiovascular system are known to be the major cause of morbidity and mortality worldwide (Lavie et al., 2019), and it seems to have greater effects on the elderly.

Therefore, with the aging process, the cardiovascular functional decline is significant (Ogliari et al., 2015) and the maintenance and improvement of this system are extremely important for the elderly's organic integrity (Pichot et al., 2005). The cardiovascular system is driven by autonomic and hemodynamic actions, where in an integrated manner it provides the functional efficiency of this system (Benda et al., 2015). However, the cardiorespiratory system is extremely important not only in the aging process but also in cardiovascular potentiality and efficiency, which can suffer significant functional reductions (Chaves et al., 2008). Thus, the cardiovascular and cardiorespiratory systems are linked to the health of the elderly and require attention in the conditional improvement of their actions (Madssen et al., 2014).

To maintain and enhance cardiovascular and cardiorespiratory functions in elderly people, the strategy seems to promote an increased aerobic profile (e.g., maximal oxygen uptake—VO_2max_). Previous studies have reported enhances in VO_2max_ reduce the risk of death from cardiovascular and cardiorespiratory events (±15%) (13). Studies report that the harmful physiological effects in the elderly are not related to aging, but to lifestyle habits, such as a lack of regular physical activity (Inouye et al., 2018). Pichot et al. (2005) mentions that aging is not a limiting factor of the autonomic nervous system. Thus, the improvement in physical conditioning is related to cardiovascular and cardiorespiratory efficiency, especially in the face of aging (Fletcher et al., 2018; Lavie et al., 2019).

Therefore, to improve the aerobic profile, specific training is necessary and interval training (IT) seems to promote positive adaptations (Astorino and Schubert, 2014). It has been suggested to improve the maximal aerobic profile in the elderly (Nemoto et al., 2007; Molmen et al., 2012) and to improve autonomic and hemodynamic modulation (Pichot et al., 2005), thus consolidating an improvement of the cardiovascular system (Rakobowchuk et al., 2013). Recently, high-intensity interval training (HIIT) has shown important results with VO_2max_ and improved cardiovascular system (Taylor et al., 2019). These are important health markers (Frazão et al., 2016; Cabral-Santos et al., 2017). Other studies have used HIIT in interventions with different populations and achieved positive results on cardiorespiratory (Castro et al., 2019), cardiometabolic (Fisher et al., 2015; Kong et al., 2016), cardiovascular, and psychological effects (Shepherd et al., 2015). In addition, regarding IT intensities, studies have focused more on the application of high intensity. However, there are still few studies concerning IT with the elderly population. Some have shown that IT is a relevant intervener in cardiovascular (Bertani et al., 2018) and cardiorespiratory (Rognmo et al., 2004) variables in the elderly.

The purpose of the present review is to synthesize findings of the cardiovascular effects concerning healthy elderly subjects. For this, the following variables were analyzed: resting HR, SBP, DBP, MBP, HRV, BA, and VO_2max_.

## Methods

### Literature Search

This systematic review was designed and reported according to the recommendations of the PRISMA guidelines (Liberati et al., 2009) and with the proposed MOOSE report (Meta-analysis of Observational Studies in Epidemiology) (Stroup et al., 2008).

A systematic literature search was conducted through May 2019, using the following databases: PubMeb, Medline, and Sport Discus. Search terms were defined according to population (elderly) and intervention (interval training), based on previous systematic reviews on the field. The following search strategies, Medical Subject Headings (MeSH), and Boolean operators were considered: “Interval Training and Elderly and Interval Training and Aging and Baroreflex Sensitivity” OR “Interval Training and Aging and Blood Pressure” OR “Interval Training and Aging and Heart Rate Variability” OR “Interval Training and Elderly and Baroreflex Sensitivity and Interval Training and Elderly and Heart Rate Variability.” Four researchers who reached consensus in case of disagreement performed these procedures.

We included studies that used IT as an intervention protocol (even if compared to other types of intervention), using a sample of healthy individuals aged 60 years or older, who investigated at least one variable (even indirectly, not thus the main objective of the study) that was of interest to our review. Articles that had no association with the purpose of this study, articles that had protocols that did not fit the selection, and that did not mention the description of the protocols, methodology, and sampling were excluded. Pilot and review studies were also excluded.

After merging search results and discarding duplicates, two researchers independently screened titles and abstracts in order to identify relevant studies. Full-text articles of the included reports were retrieved and independently assessed for eligibility by the two researchers according to the previously described criteria. A consensus meeting was performed in case of disagreement regarding any report and a third researcher completed the decision when required. When it was not possible to retrieve full-text articles, authors were contacted using email and Research Gate in order to provide the required report. After three failed attempts to obtain a response from the respective authors, the report was excluded from analysis. Some reports were seemingly published based on data from the same trials. Corresponding authors were contacted in order to confirm whether these reports were actually produced from different trials or not.

### Eligibility Criteria and Study Selection

The eligibility criteria for study inclusion were established according to the PICOS strategy:

- Population: participants must be healthy elderly.
- Intervention: any sort of acute or regular interval training intervention aimed at increasing cardio protection in elderly individuals.
- Comparison/Control: interval training interventions must be compared to another type of cardiorespiratory training, waiting control groups, or treatment-as-usual.
- Outcomes: outcomes were measures related to cardio protection in elderly (HR, HRV, SBP, DBP, MAP, BA, and VO_2max_).
- Study Design/Type: Original article. Randomized controlled trials, using either cross-over or parallel group designs, comparing an intervention(s) encompassing interval training with a group of another type of cardiorespiratory training, waiting control groups, or treatment-as-usual.

We included studies that used IT as an intervention protocol (even in comparison with other types of intervention), using a sample of healthy individuals aged 60 or over (free of any functional limitations or medical conditions) and that investigated at least one variable (even if indirectly, therefore not being the main objective of the study) of interest for our analysis. For organizational determination, only original studies published between 1990 and 2019 in English were included. Screening was performed by reading the title, summary, and, when necessary, reading in full for a more detailed assessment. Then, an eligibility process was carried out by reading all the articles in full. The reference lists studied were revised to identify other studies. Finally, after the entire eligibility process, articles for systematic review were included. Studies based on methodological quality and those that did not fit the research objectives were excluded.

### Risk of Bias in Individual Studies

To assess the risk of bias in individual studies, the researchers carried out an analysis of the methodological quality of the studies. The assessment tool for the selected studies was carried out using the PEDro scale (Center for Evidence-Based Physiotherapy, 2019). The PEDro scale is considered an appropriate tool in systematic reviews for qualitative analysis of quantitative studies. The method consists of component classifications for the following categories: selection criteria, equation between groups, data collection methods, and outcome factors. The components were classified at 0 (not identified) and 1 (identified). Studies with PEDro scores between 6 and 10 points, 4 and 5 points, and 0 and 3 points were considered high, moderate, and low quality, respectively. All disagreements regarding rating of PEDro scores were resolved by a consensus discussion between the reviewers.

## Results

### Study Selection

**Figure 1:**
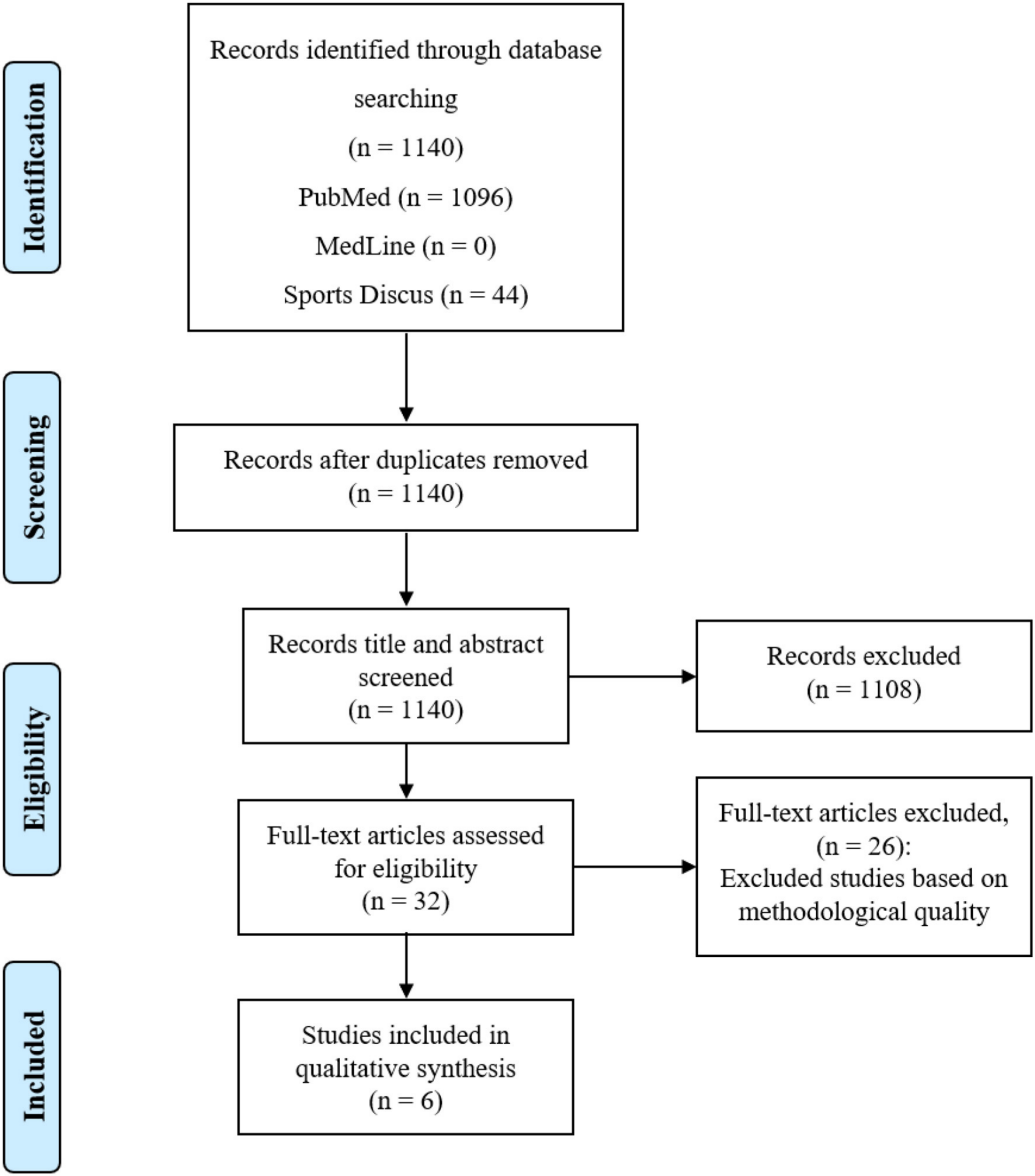
Flow diagram of information on the different phases of the systematic review.

After using the keywords, 1,140 articles were identified. However, in the article screening process, 1,108 articles were excluded by checking their titles and abstracts. Finally, 32 studies were selected for full reading. After eligibility, 26 were eliminated because they did not contain a methodology according to the purpose of this review, with six studies included for the final analysis. The whole study selection process is shown in the PRISMA flow diagram in Figure 1.

### Study Quality

The mean PEDro score for the studies included in the review was 4.8 ± 1.3 points with a range of 3–6 points (Table 1). According to the quality criteria set, the average quality of the studies included in this review is therefore moderate. Moreover, there was not a high degree of variation in quality between studies. All studies met the eligibility criterion (PEDro scale question 1) and outcome measures. Three studies (Nemoto et al., 2007; Lepretre et al., 2009; Adamson et al., 2019) performed a crossover randomized design (PEDro scale question 2). None of the studies concealed criteria (PEDro scale question 3). Four studies (Ahmaidi et al., 1998; Nemoto et al., 2007; Lepretre et al., 2009; Adamson et al., 2019) showed similarity between groups (PEDro scale question 4). None of the studies had blinded methodological criteria (PEDro scale questions 5, 6, and 7). All studies showed results in more than 85% of the sample (PEDro scale question 8) and all studies met criteria 9, related to the intervention condition (PEDro scale question 9). Five studies (Ahmaidi et al., 1998; Nemoto et al., 2007; Lepretre et al., 2009; Molmen et al., 2012; Adamson et al., 2019) showed statistical comparisons between groups (PEDro scale question 10) and provided point measures and measures of variability (Pichot et al., 2005; Nemoto et al., 2007; Lepretre et al., 2009; Molmen et al., 2012; Adamson et al., 2019) (PEDro scale question 11).

**Table 1:** PEDro score of methodological quality for included studies.

**Reference**	**1**	**2**	**3**	**4**	**5**	**6**	**7**	**8**	**9**	**10**	**11**	**Totality**
Ahmaidi et al. (1998)	1	0	0	1	0	0	0	1	1	1	0	4
Pichot et al. (2005)	1	0	0	0	0	0	0	1	1	0	1	3
Nemoto et al. (2007)	1	1	0	1	0	0	0	1	1	1	1	6
Lepretre et al. (2009)	1	1	0	1	0	0	0	1	1	1	1	6
Molmen et al. (2012)	1	0	0	0	0	0	0	1	1	1	1	4
Adamson et al. (2019)	1	1	0	1	0	0	0	1	1	1	1	6

*0: Did not meet criterion; 1: Met the criterion*.

### Study Characteristics

The summary of articles, in Table 2, was based on a structured questionnaire that considered the following items: Authors, year of publication, sampling (amount, gender, and age), training protocols, dependent variable, and results (Neto et al., 2017).

**Table 2:** Selected studies that investigated interval training in cardioprotective variables in the elderly.

**References**	**Sampling (groups, *N*, gender, age)**	**Training protocols**	**Duration**	**Results**
Ahmaidi et al. (1998)	*N* = 22 Mens GE: 11 (62.7 + 1.4) GC: 11 (61.7 + 1.9)	In Natural way IT 10 ' Heating 1' St./1' Rec.; 2 ' St./1' Rec. 4' St./1' Rec.; 10' St./3' Rec. Intensity: St.: HR-VT_H_/Rec.: 20 BPM less HR-VT_H_ 10' Deceleration	12 weeks/2 x week	Improved relative VO_2max_. There was no difference in absolute VO_2*max*_
Pichot et al. (2005)	*N* = 11 Mens GE: M: 11 (73.5 ± 4.2) GC: NR	In Cycle ergometer IT 9 x 4' 65% HR 1' 85% HR	14 weeks/4 x week	Improved HR, HRV, BA and VO_2max_ relative. There was no difference in HRV (PNN50) SBP, DBP, and MAP
Nemoto et al. (2007)	*N* = 28 Mens/111 Womens GE 1: M: 11 (67 + 4)/W: 31 (64 + 6) GE 2: M: 8 (67 + 5)/W: 43 (62 + 5) GC: M: 9 (66 + 5)/W: 37 (62 + 6)	In Natural Way IT (HIIWT) 5 (or more) x 3' 40% VO_2peak_ 3' 70–85% VO_2peak_	5 months/2–4 weeks	Improved HR (womens), SBP and DBP (men and women).
Lepretre et al. (2009)	*N* = 16 Mens/19 Womens GE: M: 16 (64 ± 3.7) / W: 19 (65.5 ± 5.4) GC: NR	In Cycle ergometer IT 6 x 4' 1° VT_H_ 1' 2° VT_H_	9 weeks/2 x weeks	Improved VO_2max_ relative. There was no difference in SBP and DBP.
Molmen et al. (2012)	*N* = 21 Mens/6 Womens GE: M: 10/W: 6 (73 ± 3) GC1: M 10 (23 ± 2) GC2: M: 11 (74 + 2)	In Treadmill IT 10' Heating ~60% HR_max_ 4x 4' 90–95% HR_max_ 3' 60–70% HR_max_ 3' Deceleration	12 weeks/3 x weeks	Improved HR, SBP, DBP and VO_2max_ relative.
Adamson et al. (2019)	*N* = 9 Mens/8 Womens GE: M: 6/W: 4 (66 ± 4) GC: M: 3/W: 4 (66 ± 2)	In Cycle ergometer IT (SIT) 6 a 11 x 6” All Out 30” Passive Rec. (or HR below 120 BPM)	10 weeks/2 x weeks	Improved SBP (mens and womens) and DBP (mens). There was no difference in DBP (womens) and MAP (mens and womens).

*EG, Experimental Group; CG, Control Group; NR, Not done; IT, Interval Training; HIIWT, High Intensity Interval Walking Training; SIT, Sprint Interval Training; St., Stimulus; Rec., Recovery; HR-VT_H_, Heart Rate at Ventilatory Threshold; HR_max_, Maximum Heart Rate; VO_2_peak, Peak Oxygen Consumption; VT, Ventilatory Threshold; VO_2max_, Maximum Oxygen Consumption; HRV, Heart Rate Variability; BA, Baroreflex Activity; HR, Resting Heart Rate; SBP, Systolic Blood Pressure; DBP, Diastolic Blood Pressure; MAP, Mean Arterial Pressure*.

In these selected studies, the findings were from publications from 1998 (Ahmaidi et al., 1998) to 2019 (Adamson et al., 2019). Regarding sampling, 251 individuals were found, 107 men and 144 women. However, two studies used only men in their interventions (Ahmaidi et al., 1998; Pichot et al., 2005) and the other four studies used men and women in their analyses (Nemoto et al., 2007; Lepretre et al., 2009; Molmen et al., 2012; Adamson et al., 2019). No female-only studies were found, and two studies used active individuals (Ahmaidi et al., 1998; Pichot et al., 2005) and four studies included inactive individuals (Nemoto et al., 2007; Lepretre et al., 2009; Molmen et al., 2012; Adamson et al., 2019). The sample size in the selected studies ranged from 11 (Pichot et al., 2005) to 139 individuals (Nemoto et al., 2007).

Regarding the body composition of the participants in the selected studies, two studies did not present these data (Ahmaidi et al., 1998; Nemoto et al., 2007). Pichot et al. (2005) presented weight 81 ± 12.0 (kg) and BMI 28.4 ± 4.0 (kg/m^2^) but did not present data on height. Lepretre et al. (2009) presented weight 82.9 ± 11.2 and 65.0 ± 9.9 (kg) and height 177 ± 5.7 and 161.5 ± 5.4 (cm) for men and women, respectively, but did not present BMI. Molmen et al. (2012) used three groups, two with elderly people and one with young people (control). Regarding the elderly, one group had values of weight 76.5 ± 9.4 (kg) and BMI 25.0 ± 2.5 (kg/m^2^) and another with weight of 74.5 ± 8.3 (kg) and BMI 23.0 ± 1.9 (kg/m^2^). In the group with young people, the weight was 76.0 ± 16.0 (kg) and BMI 22.7 ± 3.1 (kg/m^2^). They did not provide data on height. Adamson et al. (2019) presented for the experimental group weight 77 ± 1.3 (kg), height 169 ± 9 (cm), and BMI 26.9 ± 3.5 (kg/m^2^) and for the control group weight of 70 ± 1.3 (kg), height 1.64 ± 10, and BMI 25.9 ± 3.3 (kg/m^2^).

The findings of the present study identified different protocols for interventions: IT (Ahmaidi et al., 1998; Pichot et al., 2005; Lepretre et al., 2009; Molmen et al., 2012), Sprint Interval Training (SIT) (Adamson et al., 2019), and the High Intensity Interval Walking Training (HIIWT) (Nemoto et al., 2007). With the advances and popularization of the interval training using different intensities of effort, it was necessary to elaborate on different nomenclatures for this method. The commonly used IT is represented by physical exertion with cyclic and repetitive exercises, alternating stimulus periods based on some physiological parameters, which may be below the anaerobic threshold, lower than 85% of maximum heart rate (HRmax) or 85% of VO_2max_ (Buchheit and Laursen, 2013). Therefore, it is a method that provides significant physiological adaptations in improving fitness (Billat, 2001). The SIT is conducted by short (5 to 60”) stimuli with markedly high intensities that may be a high intensity (90% VO_2max_ or above the 2nd ventilatory threshold), maximal (100% VO_2max_), supramaximal (>100% VO_2max_), or subjectively, commonly called “all-out” (30). HIIWT is an IT protocol that uses walking as a mechanical rhythm. However, even though it is not common among IT applications, HIIWT can be an important strategy for the elderly unable to perform an activity with a high level of impact and motor demands (Oliveira et al., 2012). Regardless of the methodological variation, all these protocols have characteristics for a predominance of aerobic metabolism (Gaitanos et al., 1993; Billat, 2001).

The different IT protocols were conducted in order to obtain improvements in cardio protective variables in the elderly. Ahmaidi et al. (1998) introduced a varied sequence of stimuli with HR related to ventilatory threshold (VT) and recovery in HR of 20 beats below that obtained in VT, totaling 43 min of activity. Pichot et al. (2005) applied six sets of 4 min at 65% HRmax and 1 min at 85% HRmax for 45 min. Nemoto et al. (2007) did not fix a number of interval series, performing five or more blocks of 3 min at 40% VO_2peak_ with 3 min at 70–85% VO_2peak_, establishing a volume of at least 30 min. Lepretre et al. (2009) used VT as a parameter to control IT intensities for 30 min, with six series of 4 min in the 1st with 1 min in the 2nd. Molmen et al. (2012) performed 28-min sessions with 4-min stimuli at 90–95% HRmax and 3-min recovery at 60–70% HRmax. Adamson et al. (2019) used a protocol with 6 to 11 sets of 6 s at maximum intensity (all-out) for 30 s of passive recovery or when heart rate below 120 BPM was reached. Additionally, considering the methodological aspects of IT, the present study identified that all selected articles used high-intensity stimuli.

Regarding the location, equipment, and time of intervention, two studies performed the training in the natural way (Ahmaidi et al., 1998; Nemoto et al., 2007) and four were conducted in laboratories using cycle ergometers (Pichot et al., 2005; Lepretre et al., 2009; Adamson et al., 2019) or treadmills (Molmen et al., 2012). All selected studies applied chronic interventions ranging from 9 (Lepretre et al., 2009) to 20 weeks (Nemoto et al., 2007). To determine the statistical analysis, all selected studies had a significance level of *p* < 0.05. Regarding the hemodynamic, autonomic, and cardiorespiratory responses resulting from the different IT protocols, four studies investigated the measurement of HR (Ahmaidi et al., 1998; Pichot et al., 2005; Nemoto et al., 2007; Molmen et al., 2012), five studies verified the behavior of SBP and DBP (Pichot et al., 2005; Nemoto et al., 2007; Lepretre et al., 2009; Molmen et al., 2012; Adamson et al., 2019), and two studies measured MAP (Pichot et al., 2005; Adamson et al., 2019). Regarding autonomic control, only one study investigated this performance through BA and HRV (Pichot et al., 2005). In the cardiorespiratory condition, four studies evaluated the influence of IT on VO_2max_ (Ahmaidi et al., 1998; Pichot et al., 2005; Lepretre et al., 2009; Molmen et al., 2012). However, only one study investigated hemodynamic, autonomic, and cardiorespiratory variables (Pichot et al., 2005).

On measurement procedures for HR, a study (Pichot et al., 2005) used the Holter method and two studies (Nemoto et al., 2007; Molmen et al., 2012) used the electrocardiogram method. Regarding Blood Pressure (BP), a study (Pichot et al., 2005) used the measurement using the volume clamp method on the fingers, one study used the auscultatory method (Nemoto et al., 2007), one study used the electrocardiogram method (Molmen et al., 2012), one study (Adamson et al., 2019) used an automatic device, and only one study (Lepretre et al., 2009) did not mention which method for BP was used, possibly because it did not present BP results in an indirect way in the study, not being the main target of the study. Regarding BA, only one study analyzed this variable (Pichot et al., 2005) and used the sequence and cross-spectral analysis method using an electrocardiogram. For HRV, only Pichot et al. (2005) analyzed and was using the Holter method. Finally, for the analysis of VO_2max_, all studies that performed this evaluation (Ahmaidi et al., 1998; Pichot et al., 2005; Lepretre et al., 2009; Molmen et al., 2012) used the method with incremental testing gases. Two studies were carried on a treadmill (Ahmaidi et al., 1998; Molmen et al., 2012) and two studies on cycle ergometers (Pichot et al., 2005; Lepretre et al., 2009). Ahead, the results for each variable on the effects of IT on different protocol formats will be displayed.

## Outcome Measures

### Resting Heart Rate (HR)

HR is extremely important in clinical evaluation and is related to cardiovascular disorders (Schneider et al., 2018). Several factors can modify the behavior of this variable, one of which is aging (Ogliari et al., 2015). Regarding the HR of the selected studies, some important results about this variable are presented. Pichot et al. (2005) obtained significant results in HR, being 71.9 ± 9.9 BPM at baseline and 67.2 ± 11.4 BPM post-intervention (*p* < 0.001). Nemoto et al. (2007) achieved interval HR improvements for women (81 ± 2 to 78 ± 1 BPM, *p* < 0.05). More expressively, Molmen et al. (2012) demonstrated a reduction of 10 beats (*p* < 0.01) after intervention with IT.

### Systolic, Diastolic and Mean Blood Pressure (SBP, DBP, and MAP)

BP is a feature that changes significantly in the aging process and one of the main factors is the stiffness caused in the arterial structures (Figueroa et al., 2010) and the loss of functional efficiency of the elderly cardiovascular system (Deley et al., 2009). Thus, regarding SBP, some related studies showed significant results after their interventions. In the study by Nemoto et al. (2007) there was a reduction of 10 mmHg for men (146 ± 2 to 136 ± 2, *p* < 0.001) and 8 mmHg for women (140 ± 3 to 132 ± 2, *p* < 0.001). Molmen et al. (2012) were able to reduce SBP by 12% (143 ± 15.0 to 126 ± 8.5, *p* < 0.05). Adamson et al. (2019) achieved improvements of 4 mmHg for men (136 ± 13 to 122 ± 9, *p* < 0.05) and 10 mmHg for women (141 ± 13 to 131 ± 6, *p* < 0.05). Pichot et al. (2005) failed to observe significant differences in SBP (111.6 ± 13.9 to 111.1 ± 13.3, *p* > 0.05), as did Lepretre et al. (2009) for whom SBP reduced very discreetly for men and women (0.7% and 1.3%, respectively, *p* > 0.05). Regarding DBP, the results followed the same line. Nemoto et al. (2007) obtained a reduction in DBP for both men and women, with 87 ± 3 to 82 ± 2 (*p* < 0.05) and 85 ± 2 to 80 ± 2 (*p* < 0.001), respectively. Molmen et al. (2012) decreased by 9% (*p* < 0.01) from 80.0 ± 8.7 to 73 ± 5.0 after the interventions. Adamson et al. (2019) were able to decrease DBP by 8 mmHg for men (85 ± 5 to 77 ± 9, *p* < 0.05), but for women did not obtain significant differences (85 ± 5 to 84 ± 4, *p* > 0.05). Pichot et al. (2005) and Lepretre et al. (2009) did not achieve significant results in DBP behavior after the training period (*p* > 0.05). Regarding MAP, only two studies verified that Pichot et al. (2005) did not achieve significant differences (76.9 ± 14.9 to 77.9 ± 12.3, *p* > 0.05). On the other hand, Adamson et al. (2019) demonstrated significant reductions for both men (104 ± 9 to 94 ± 10, *p* < 0.05) and women (94 ± 8 to 88 ± 11, *p* < 0.05).

### Heart Rate Variability (HRV)

HRV is an important measure to diagnose the cardiovascular condition (Ogliari et al., 2015). Therefore, the interpretation of the acquired data is subdivided into the time domain and frequency domain with their respective indices (Laborde et al., 2017). In the time domain, the values on beats to beats (RR), PNN50, and RMSSD that are associated with the parasympathetic activity are acquired (Young and Benton, 2018). SDNN and SDANN are related to global autonomic activity. In the frequency domain, the low frequency (LF) components are analyzed, corresponding to the sympathetic and parasympathetic joint action with sympathetic predominance. The high frequency (HF) component indicates parasympathetic performance and the LF/HF ratio is a marker of autonomic balance (Vanderlei et al., 2009). Given the studies selected for the present review, only one study investigated HRV. Pichot et al. (2005) demonstrated relevant results on HRV assessment items. In time-domain analyses, the researchers in this study found a 7.7% improvement in RR values (847 ± 100 to 912 ± 133, *p* < 0.001) and a 15.4% reduction in SDNN (149 ± 45 to 126 ± 40, *p* < 0.05). Significant results were also found in the RMSSD indices (30.3 ± 7.5 to 36.4 ± 8.8, *p* < 0.01). However, for PNN50 there were no significant differences (3.52 ± 2.53 to 4.41 ± 2.79, *p* > 0.05). For frequency domain analysis, Pichot et al. (2005) also presented interesting results. For the LF (n.u.), baseline results were 62.4 ± 9.5 and 58.4 ± 11.4 after interventions (*p* < 0.05). For HF (n.u.) the results were also positive, being 37.6 ± 9.5 pre-intervention and 41.4 ± 11.4 after training (*p* < 0.05). Regarding the LF/HF measurement, there was a 19.1% reduction (2.93 ± 1.35 to 2.37 ± 1.10, *p* < 0.05).

### Baroreflex Activity (BA)

BA is a feature that demonstrates cardiovascular efficiency (Deley et al., 2009; Figueroa et al., 2010), promoting in an integrated way the modulation of hemodynamic and autonomic systems. Only one study investigated the behavior of BA (Pichot et al., 2005). In this study, we used the sequence methods that use RR behavior with SBP and cross-spectral analysis that observes the performance of low (LF) and high (HF) frequency components with BP, all expressed in ms.mmHg. The results of Pichot et al. (2005) demonstrated improvements in BA. In the sequence method, baroreflex activity increased 40% significantly from 7.0 ± 1.8 to 9.8 ± 2.1 ms.mmHg (*p* < 0.01). From these findings, 10 subjects showed improvement and only one subject reduced BA. Regarding spectral analysis, when the researchers performed the calculations through the HF, they obtained positive results increasing significantly from 6.9 ± 2.2 to 10.5 ± 3.7 ms.mm/Hg-1 (52.5%, *p* < 0.05), with eight subjects showing an increase in BA and two showing a decrease. When the evaluation was performed using the LF values as the basis of calculation, no significant differences were observed (from 5.3 ± 2.3 to 6.9 ± 3.1 ms.mmHg-1, *p* = 0.22).

### Maximum Oxygen Consumption (VO_2max_)

VO_2max_ is of paramount importance for the elderly because in the aging process there is a reduction in the efficiency of cardiovascular function due to the decrease in cardiorespiratory fitness, and in the elderly VO_2max_ can reduce 10% in sedentary and 5% in assets (Oliveira et al., 2012). Studies have found relevant results on VO_2max_ after interventions. Ahmaidi et al. (1998) obtained a significant increase in absolute and relative VO_2max_, from 1.77 to 2.11 l.min^−1^ (*p* < 0.01) and from 25.42 to 30.63 ml.kg.min^−1^ (*p* < 0.01), respectively. Pichot et al. (2005) also demonstrated significant differences. The authors found an 18.6% increase in relative VO_2max_ values (26.84 ± 4.38 to 31.82 ± 5.15 ml.kg.min^−1^, *p* < 0.01) and observed relevant absolute VO_2max_ results (*p* < 0.01), however the exact values were not exposed. Lepretre et al. (2009) found a 14.9% increase in relative VO_2max_ for men (27.0 ± 5.1 to 29.9 ± 4.5 ml.kg.min^−1^, *p* < 0.05) and 14.5% for women (18.6 ± 3.6 to 21.1 ± 3.7 ml. kg.min^−1^, *p* < 0.05). However, these authors did not observe intergroup differences (*p* = 0.237). Molmen et al. (2012) conducted the investigation on VO_2max_ of men (*N* = 10) and women (*N* = 6) and found significance in their results. In the analyses for men, VO_2max_ changed from 35.0 ± 5.0 to 39.0 ± 7.2 ml.kg.min^−1^ (*p* < 0.01) and, when considering the group containing men and women, a 15% increase in VO_2max_ was found after the interventions, from 32.5 ± 5.5 to 37.0 ± 6.1 ml.kg.min^−1^ (*p* < 0.01). Therefore, it is concluded that, as presented in the investigated studies, IT is an important intervener on VO_2max_ with its different protocol variations.

## Discussion

This study aimed to verify the efficiency of IT on cardiovascular and cardiorespiratory variables, more specifically HRR, HRV, SBP, DBP, MAP, BA, and VO_2max_. These directly act on the cardio protection functionality. We identified 1,140 articles, but only six studies fit our purpose (Ahmaidi et al., 1998; Pichot et al., 2005; Nemoto et al., 2007; Lepretre et al., 2009; Molmen et al., 2012; Adamson et al., 2019). From these results, it is valid to state that there is a significant limitation of studies related to the elderly population submitted to IT. Thus, the scarcity of studies related to the theme of the present review corroborates those of Ferreira et al. (2017) who aimed to select studies that intervened with aerobic training in HRV in the elderly and found only seven studies for a systematic review. Given our findings, it is necessary to state that the selected studies obtained positive results in cardiovascular and cardiorespiratory variables. Regarding the cardiovascular system, the selected studies presented important results on the IT responses on hemodynamic (Ahmaidi et al., 1998; Pichot et al., 2005; Nemoto et al., 2007; Lepretre et al., 2009; Molmen et al., 2012; Adamson et al., 2019) and autonomic variables (Pichot et al., 2005). Regarding VO_2max_, studies have also shown improvements after distinct interventions using IT (Ahmaidi et al., 1998; Pichot et al., 2005; Lepretre et al., 2009; Molmen et al., 2012). This demonstrates the potentiality of IT in the main cardiovascular and cardiorespiratory variables, these being HR, HRV, SBP, DBP, MAP, BA, and VO_2max_ that, in an integrated manner, act directly and decisively on the efficiency and balance of the cardiovascular system.

HR is an important predictor of cardiovascular health (Ogliari et al., 2015) and our findings reinforce the hypothesis that IT is efficient in HR behavior (Pichot et al., 2005). Studies have improved from 3 beats (Nemoto et al., 2007) to 10 beats (Molmen et al., 2012) in the elderly surveyed. About HRV, only one study (Pichot et al., 2005) investigated this variable that indicates the level of cardiovascular health and possible risks of this system (Young and Benton, 2018; Geus et al., 2019). However, in the study by Pichot et al. (2005) improvements in time between one cardiac cycle and another by 7.7% (RR) were observed. In the time domain analysis, improvements were found in parasympathetic performance indices (RMSSD). Regarding the PNN50 index (%) that is related to parasympathetic reactivation, no statistical differences were found. However, they found a reduction in values on the SDNN index that is related to both autonomic actions with a sympathetic prevalence. Also, the reduction in this index is a consequence of the parasympathetic system's role in promoting balance and cardiovascular control. In the frequency domain, all indices have been improved. In the LF (sympathetic-parasympathetic) there was a significant reduction, demonstrating a positive parasympathetic performance. Regarding HF, which is related to the parasympathetic system, there was a significant increase. Consolidating IT efficiency over HRV in the elderly, Pichot et al. (2005) found a 19.1% reduction in LF/HF values, demonstrating that the intervention was efficient in autonomic modulation.

Regarding BP, one of the factors that makes this functionality worse is arterial stiffness caused by the aging process (Figueroa et al., 2010), thus reflecting the functional inefficiency and impairing the cardiovascular system (Deley et al., 2009). However, this review has shown that IT is a great intervention in hemodynamic improvement. Of the five selected studies investigating hemodynamic functionality, three obtained positive results after their interventions in SBP and DBP (Nemoto et al., 2007; Molmen et al., 2012; Adamson et al., 2019). Pichot et al. (2005) and Lepretre et al. (2009) did not observe statistical differences in these measures. Regarding MAP, Pichot et al. (2005) did not present significant differences, but Adamson et al. (2019) achieved positive results. However, we can say that IT is an efficient methodology for hemodynamic improvement. Regarding the integration of the autonomic and hemodynamic system, BA is the indicator of cardiovascular condition on this aspect. However, only Pichot et al. (2005) evaluated this measure using HRV and SBP and found positive results after interventions. The researchers used the sequence and spectral method and found significant differences in both (40% and 52%, respectively), demonstrating that IT is an excellent method for improving this measure of great importance in the control and balance of the cardiovascular system (Deley et al., 2009).

In VO_2max_ all studies that performed this measure obtained positive results (Ahmaidi et al., 1998; Pichot et al., 2005; Lepretre et al., 2009; Molmen et al., 2012). The increases were from 15 to 18.6%, which are of great value, considering that in the aging process the reduction of aerobic capacity can be 10% for sedentary individuals and 5% for those who are active (Oliveira et al., 2012). Among these studies, all directly measured VO_2max_ (gas analyzers), determining the reliability of the data obtained and that IT is efficient in improving cardiorespiratory capacity, which is an important determinant of cardiovascular health in the elderly (Kodama et al., 2009).

The mechanisms related to hemodynamic, autonomic, and cardiorespiratory improvements may be due to hormonal, neural, metabolic, and structural changes caused by different IT stimuli. In the hemodynamic condition, IT balances the renin-angiotensin system allowing for better behavior of both systolic and diastolic BP (Pichot et al., 2005). Other beneficial factors of IT are reduced arterial stiffness and improved endothelial function and plasma volume (Molmen et al., 2012; Adamson et al., 2019). In this way, the BP behavior becomes better both in the exercise condition and at rest. HR refers to the reduction caused naturally due to aging. But it is suggested that IT acts on improving cardiac conditioning by interfering with atrioventricular efficiency (Molmen et al., 2012). Regarding neural control over cardiovascular condition, it is speculated that repeated series of effort and recovery promotes stimuli capable of altering the activity of the autonomic nervous system, which chronically may improve neuro-cardial functioning being signaled by a higher HRV and better BA, and these features are considered as excellent markers of the integration of the autonomic and hemodynamic system (Pichot et al., 2005). Consequently, the higher the ratio, the better the cardiovascular conditional status. Pichot et al. (2005), through the analysis of HRV and BA, observed better parasympathetic responses after IT intervention. This suggests that advanced age is not a limiting factor for adaptations in the autonomic nervous system, more specifically in a population with a mean age of 73 years. Regarding cardiorespiratory fitness, the aging process promotes considerable decline for both sedentary and active individuals (Oliveira et al., 2012). However, IT can enable a significant increase in VO_2max_ for the elderly public. This improvement may occur due to higher capillary and mitochondrial density (Nemoto et al., 2007). Other factors related to increased VO_2max_ in the elderly is that IT promotes more efficiency in the relationship between transport and consumption of O_2_ by higher amounts of circulating hemoglobin and better muscle condition (Lepretre et al., 2009). However, it seems that the hemodynamic, autonomic, and cardiorespiratory improvements in the elderly are triggered in an integrated manner.

All studies applied IT with different protocols in terms of stimulus and recovery. There is a difficulty in determining which type of interval (intensive or extensive) is most productive. In the present study, Pichot et al. (2005) demonstrated relevant results with an extensive feature protocol. However, Adamson et al. (2019), using an intensive protocol, also achieved great results. The important thing is to identify which level of conditioning is needed for correct and effective application of the different IT protocols. For the elderly, IT can be a great option for prescription variation, being an activity with greater motivation potential. In addition, the exposure to intensity is shorter, which can preserve the elderly from tissue and systemic overloads. In this review, only one study positively described adherence and that no individual was injured (Ahmaidi et al., 1998).

Concerning the elderly public, more information is needed to further materialize the data obtained by this review. Our findings demonstrate that there is a need for further investigation of cardio protective variables on IT intervention with the elderly, thus mitigating the existing gaps on this topic. However, the selected studies showed positive results in the evaluated measures. It is suggested that these findings are dependent on a multifactorial performance, such as stimulus time, recovery, intervention, elderly conditioning level, and other methodological factors. Thus, intensity is not the only factor that determines hemodynamic, autonomic, and cardiorespiratory changes.

## Conclusion

Interval training shows an efficient and non-medicated method that can be used to improve the cardiovascular health of the elderly population. Of the six selected studies, different results were possibly found due to the methodological approach applied by each research, but even with some discrete results, IT may be included in the prescription of aerobic training for the elderly in order to obtain conditional improvements in the cardiovascular system, thus being an important clinical intervention for this audience.

## Data Availability Statement

The raw data supporting the conclusions of this article will be made available by the authors, without undue reservation, to any qualified researcher.

## Author Contributions

LS, SM, JN, and JV conceptualized the project. AR, NR, YC, and JS performed the literature review. LS, FS, AB, and EM wrote the first draft of the manuscript. SM, JN, JV, and HB critically reviewed the manuscript regarding their areas of expertise. All authors contributed to the article and approved the submitted version.

## Conflict of Interest

The authors declare that the research was conducted in the absence of any commercial or financial relationships that could be construed as a potential conflict of interest.

## Abbreviations

PRISMA: Preferred Reporting Items for Systematic reviews and Meta-Analyses
HR: Heart Rate
SBP: Systolic Blood Pressure
DBP: Diastolic Blood Pressure
MBP: Mean Arterial Pressure
HRV: Heart Rate Variability
BA: Baroreflex activity
VO_2max_: Maximal Oxygen Uptake
ACSM: American College of Sports Medicine
AHA: American Heart Association
IT: Interval Training
HIIT: High-Intensity Interval Training.
